# Methyl 3,5-dibromo-2-diacetyl­amino­benzoate

**DOI:** 10.1107/S1600536811034568

**Published:** 2011-08-27

**Authors:** Jerry P. Jasinski, James A. Golen, A. S. Praveen, H. S. Yathirajan, B. Narayana

**Affiliations:** a Department of Chemistry, Keene State College, 229 Main Street, Keene, NH 03435-2001, USA; b Department of Studies in Chemistry, University of Mysore, Manasagangotri, Mysore 570 006, India; c Department of Studies in Chemistry, Mangalore University, Mangalagangotri, 574 199, India

## Abstract

The title methyl benzoate compound, C_12_H_11_Br_2_NO_4_, consists of an *ortho*-substituted diacetyl­amino group and *meta*-substituted Br atoms. The crystal packing is stabilized by weak inter­molecular C—H⋯O inter­actions.

## Related literature

For the use of halogenated benzoates to stimulate the microbial dechlorination of polychlorinated biphenyls, see: Deweerd & Bedard (1999 ▶). For related structures, see: Gowda *et al.* (2008 ▶); Saeed *et al.* (2010 ▶); Yathirajan *et al.* (2007 ▶). For bond lengths, see Allen *et al.* (1987 ▶).
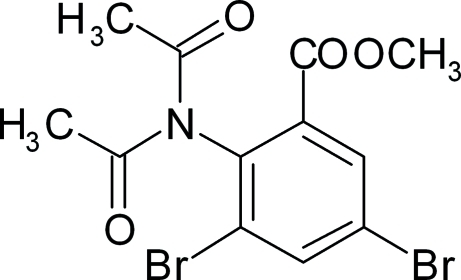


## Experimental

### 

#### Crystal data

C_12_H_11_Br_2_NO_4_
*M* *_r_* = 393.04
Triclinic, 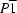

*a* = 7.6386 (8) Å
*b* = 8.8870 (6) Å
*c* = 10.8691 (8) Å
α = 78.186 (6)°
β = 76.155 (7)°
γ = 82.750 (7)°
*V* = 698.91 (10) Å^3^
*Z* = 2
Mo *K*α radiation
μ = 5.81 mm^−1^
*T* = 173 K
0.24 × 0.20 × 0.18 mm

#### Data collection

Oxford Diffraction Xcalibur Eos Gemini diffractometer
Absorption correction: multi-scan (*CrysAlis RED*; Oxford Diffraction, 2010 ▶) *T*_min_ = 0.336, *T*_max_ = 0.421
5598 measured reflections
2864 independent reflections
2186 reflections with *I* > 2σ(*I*)
*R*_int_ = 0.024

#### Refinement

*R*[*F* ^2^ > 2σ(*F* ^2^)] = 0.033
*wR*(*F* ^2^) = 0.070
*S* = 1.00
2864 reflections
175 parameters
H-atom parameters constrained
Δρ_max_ = 0.43 e Å^−3^
Δρ_min_ = −0.55 e Å^−3^

### 

Data collection: *CrysAlis PRO* (Oxford Diffraction, 2010 ▶); cell refinement: *CrysAlis PRO*; data reduction: *CrysAlis RED* (Oxford Diffraction, 2010 ▶); program(s) used to solve structure: *SHELXS97* (Sheldrick, 2008 ▶); program(s) used to refine structure: *SHELXL97* (Sheldrick, 2008 ▶); molecular graphics: *SHELXTL* (Sheldrick, 2008 ▶); software used to prepare material for publication: *SHELXTL*.

## Supplementary Material

Crystal structure: contains datablock(s) global, I. DOI: 10.1107/S1600536811034568/dn2715sup1.cif

Structure factors: contains datablock(s) I. DOI: 10.1107/S1600536811034568/dn2715Isup2.hkl

Supplementary material file. DOI: 10.1107/S1600536811034568/dn2715Isup3.cml

Additional supplementary materials:  crystallographic information; 3D view; checkCIF report

## Figures and Tables

**Table 1:** Hydrogen-bond geometry (Å, °)

*D*—H⋯*A*	*D*—H	H⋯*A*	*D*⋯*A*	*D*—H⋯*A*
C1—H1*A*⋯O4^i^	0.98	2.44	3.404 (4)	168
C6—H6*A*⋯O4^ii^	0.95	2.46	3.237 (4)	140

Symmetry codes: (i) 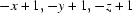; (ii) 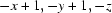.

**Fig. 1.**
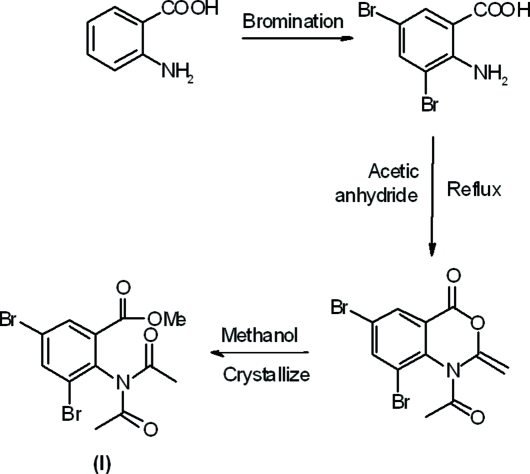
Reaction scheme of the title compound.

**Fig. 2.**
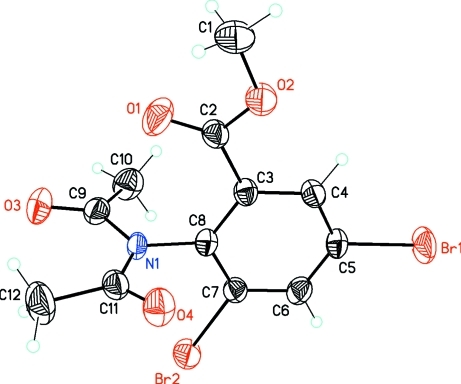
Molecular structure of the title compound showing the atom labeling scheme and 50% probability displacement ellipsoids.

## supplementary crystallographic information

### Comment

The title compound, (I), was obtained as an unexpected product in the present synthetic reaction (Fig. 1). Benzoates have wide importance in the plastics, food and pharmaceutical industries. The use of halogenated benzoates to stimulate the microbial dechlorination of poly chlorinated biphenyls is discussed (Deweerd & Bedard, 1999). The crystal structures of 4-bromophenyl benzoate (Gowda *et al.*, 2008), methyl 4-(bromomethyl)benzoate (Yathirajan *et al.*, 2007) and methyl 3,5-dibromo-4-methylbenzoate (Saeed *et al.*, 2010) have been reported. In view of the importance of benzoates, the crystal structure of title compound, (I), C_12_H_11_Br_2_NO_4_, is reported.

The title methyl benzoate compound, (I), consists of an ortho substituted N, N diacetyl group and meta substituted dibromine atoms (Fig. 2).  Crystal packing is stabilized by weak C—H···O intermolecular interactions (Table 1).

### Experimental

Preparation of 2-amino-3, 5-dibromobenzoic acid: A mixture of 2-aminobenzoic acid (25 g, 0.1822 mol) in acetic acid (50 mL) was cooled at 273 –278 K. A mixture of bromine (32.79 g, 10.5 mL, 0.1822 mol) in acetic acid (1:1 by Vol.) was added drop wise over 30 min. After addition, the mixture was stirred at 273-278 K for one hour and at room temperature for 3-4 hours. To the mixture, water (100ml) was added at 288-293 K. The solid was filtered, washed with water (50 mL x 2), and dried at 353 K for 5 hrs (Yield - 93 %).

Preparation of methyl 2-(N-acetylacetamido)-3,5-dibromobenzoate: In a 500 mL round bottomed flask, acetic anhydride (150 mL) warmed at 353 K, 2-amino-3,5-dibromobenzoic acid ( 50 g, 0.1695 mol) was added over 30 minutes. The mixture was refluxed at 411-413 K and maintained for 4 hrs, cooled to room temperature and filtered.

The crystallization was done using methanol. The title compound was obtained as an unexpected product as shown in Scheme 1. X-ray quality crystals were obtained by a slow evaporation from methanol solution (m.p.: 380-383 K).

### Refinement

All of the H atoms were placed in their calculated positions and then refined using the riding model with Atom—H lengths of 0.95Å (CH), or 0.98Å (CH_3_).  Isotropic displacement parameters for these atoms were set to 1.20-1.21 (CH) or 1.47-1.50 (CH_3_) times *U*_eq_ of the parent atom.

### Crystal data

**Table:**

C_12_H_11_Br_2_NO_4_	*Z* = 2
*M**_r_* = 393.04	*F*(000) = 384
Triclinic, *P*1	*D*_x_ = 1.868 Mg m^−^^3^
Hall symbol: -P 1	Mo *K*α radiation, λ = 0.71073 Å
*a* = 7.6386 (8) Å	Cell parameters from 2420 reflections
*b* = 8.8870 (6) Å	θ = 3.0–32.2°
*c* = 10.8691 (8) Å	µ = 5.81 mm^−^^1^
α = 78.186 (6)°	*T* = 173 K
β = 76.155 (7)°	Block, colorless
γ = 82.750 (7)°	0.24 × 0.20 × 0.18 mm
*V* = 698.91 (10) Å^3^	

### Data collection

**Table:**

Oxford Diffraction Xcalibur Eos Gemini diffractometer	2864 independent reflections
Radiation source: Enhance (Mo) X-ray Source	2186 reflections with *I* > 2σ(*I*)
graphite	*R*_int_ = 0.024
Detector resolution: 16.1500 pixels mm^-1^	θ_max_ = 26.4°, θ_min_ = 3.0°
ω scans	*h* = −9→9
Absorption correction: multi-scan (*CrysAlis RED*; Oxford Diffraction, 2010)	*k* = −11→11
*T*_min_ = 0.336, *T*_max_ = 0.421	*l* = −11→13
5598 measured reflections	

### Refinement

**Table:**

Refinement on *F*^2^	Primary atom site location: structure-invariant direct methods
Least-squares matrix: full	Secondary atom site location: difference Fourier map
*R*[*F*^2^ > 2σ(*F*^2^)] = 0.033	Hydrogen site location: inferred from neighbouring sites
*wR*(*F*^2^) = 0.070	H-atom parameters constrained
*S* = 1.00	*w* = 1/[σ^2^(*F*_o_^2^) + (0.0286*P*)^2^ + 0.0926*P*] where *P* = (*F*_o_^2^ + 2*F*_c_^2^)/3
2864 reflections	(Δ/σ)_max_ < 0.001
175 parameters	Δρ_max_ = 0.43 e Å^−^^3^
0 restraints	Δρ_min_ = −0.55 e Å^−^^3^

### Special details

**Table:**

Experimental. The compound was further characterized by 1H nmr and mass spectrum. ^1^H NMR (CDCl_3_; 400MHz): - δ 8.165 - 8.17 (d, 1H, J = 2,ArH), 8.038 8.044 (s, 1H, J = 2, ArH,), 3.87 (s, 3H, OCH_3_), 2.27 (s, 6H , (COCH_3_)_2_); ^13^C NMR ( CDCl_3_; 100 MHz): - 171.7, 163.39, 139.72, 137.9, 134.0, 131.7, 126.9, 123.3, 53.1, 26.2. Mass data: m/e: - 391 (Molecular ion peak; M+), 393(Isotope peak; M+2), 395 (Isotope peak - M+4).
Geometry. All esds (except the esd in the dihedral angle between two l.s. planes) are estimated using the full covariance matrix. The cell esds are taken into account individually in the estimation of esds in distances, angles and torsion angles; correlations between esds in cell parameters are only used when they are defined by crystal symmetry. An approximate (isotropic) treatment of cell esds is used for estimating esds involving l.s. planes.
Refinement. Refinement of *F*^2^ against ALL reflections. The weighted *R*-factor *wR* and goodness of fit *S* are based on *F*^2^, conventional *R*-factors *R* are based on *F*, with *F* set to zero for negative *F*^2^. The threshold expression of *F*^2^ > σ(*F*^2^) is used only for calculating *R*-factors(gt) *etc*. and is not relevant to the choice of reflections for refinement. *R*-factors based on *F*^2^ are statistically about twice as large as those based on *F*, and *R*- factors based on ALL data will be even larger.

### Fractional atomic coordinates and isotropic or equivalent isotropic displacement parameters (Å^2^)

**Table:**

	*x*	*y*	*z*	*U*_iso_*/*U*_eq_	
Br1	0.13746 (6)	0.87278 (3)	0.09518 (4)	0.05337 (14)	
Br2	0.30614 (5)	0.27415 (4)	−0.01150 (3)	0.04699 (13)	
O1	0.2257 (4)	0.2652 (3)	0.5162 (2)	0.0654 (8)	
O2	0.2736 (3)	0.5047 (2)	0.5174 (2)	0.0455 (6)	
O3	0.2313 (3)	−0.0469 (2)	0.3783 (2)	0.0538 (7)	
O4	0.6102 (3)	0.2554 (2)	0.2194 (2)	0.0447 (6)	
N1	0.3244 (3)	0.1887 (2)	0.2730 (2)	0.0273 (6)	
C1	0.2881 (5)	0.4583 (4)	0.6502 (3)	0.0501 (9)	
H1A	0.3009	0.5492	0.6844	0.075*	
H1B	0.3942	0.3848	0.6553	0.075*	
H1C	0.1789	0.4095	0.7009	0.075*	
C2	0.2475 (4)	0.3942 (3)	0.4608 (3)	0.0327 (7)	
C3	0.2407 (4)	0.4522 (3)	0.3230 (3)	0.0280 (7)	
C4	0.2016 (4)	0.6093 (3)	0.2801 (3)	0.0307 (7)	
H4A	0.1821	0.6801	0.3377	0.037*	
C5	0.1916 (4)	0.6603 (3)	0.1535 (3)	0.0331 (7)	
C6	0.2210 (4)	0.5630 (3)	0.0665 (3)	0.0347 (8)	
H6A	0.2134	0.6009	−0.0206	0.042*	
C7	0.2621 (4)	0.4075 (3)	0.1094 (3)	0.0296 (7)	
C8	0.2720 (4)	0.3498 (3)	0.2359 (3)	0.0276 (7)	
C9	0.1899 (4)	0.0844 (3)	0.3316 (3)	0.0355 (8)	
C10	−0.0001 (5)	0.1457 (4)	0.3313 (4)	0.0465 (9)	
H10A	−0.0798	0.0614	0.3641	0.070*	
H10B	−0.0102	0.1924	0.2431	0.070*	
H10C	−0.0359	0.2239	0.3865	0.070*	
C11	0.5116 (5)	0.1516 (3)	0.2524 (3)	0.0344 (8)	
C12	0.5843 (5)	−0.0138 (4)	0.2654 (4)	0.0577 (11)	
H12A	0.7103	−0.0208	0.2173	0.086*	
H12B	0.5118	−0.0714	0.2308	0.086*	
H12C	0.5785	−0.0575	0.3565	0.086*	

### Atomic displacement parameters (Å^2^)

**Table:**

	*U*^11^	*U*^22^	*U*^33^	*U*^12^	*U*^13^	*U*^23^
Br1	0.0831 (3)	0.02434 (17)	0.0510 (2)	0.00970 (17)	−0.0241 (2)	−0.00047 (15)
Br2	0.0767 (3)	0.03558 (18)	0.0315 (2)	−0.00369 (17)	−0.01384 (17)	−0.01074 (14)
O1	0.128 (3)	0.0388 (13)	0.0342 (14)	−0.0195 (15)	−0.0302 (15)	0.0034 (11)
O2	0.0726 (17)	0.0369 (12)	0.0323 (13)	−0.0037 (12)	−0.0202 (12)	−0.0084 (10)
O3	0.0661 (18)	0.0289 (12)	0.0581 (17)	−0.0092 (12)	−0.0070 (13)	0.0061 (11)
O4	0.0358 (14)	0.0382 (12)	0.0600 (17)	−0.0055 (11)	−0.0124 (11)	−0.0050 (11)
N1	0.0329 (15)	0.0171 (11)	0.0305 (14)	−0.0010 (10)	−0.0078 (11)	−0.0009 (10)
C1	0.067 (3)	0.057 (2)	0.034 (2)	−0.0039 (19)	−0.0207 (18)	−0.0143 (17)
C2	0.041 (2)	0.0287 (15)	0.0307 (17)	0.0017 (14)	−0.0128 (14)	−0.0078 (14)
C3	0.0289 (17)	0.0247 (14)	0.0307 (17)	0.0013 (13)	−0.0103 (13)	−0.0035 (12)
C4	0.0350 (18)	0.0253 (14)	0.0332 (17)	0.0015 (13)	−0.0101 (14)	−0.0076 (13)
C5	0.0384 (19)	0.0211 (13)	0.0383 (19)	0.0014 (13)	−0.0115 (14)	−0.0008 (13)
C6	0.041 (2)	0.0314 (15)	0.0301 (18)	−0.0020 (14)	−0.0107 (15)	0.0013 (13)
C7	0.0352 (18)	0.0265 (14)	0.0277 (16)	−0.0022 (13)	−0.0080 (13)	−0.0055 (12)
C8	0.0296 (17)	0.0225 (13)	0.0307 (17)	−0.0007 (12)	−0.0098 (13)	−0.0020 (12)
C9	0.047 (2)	0.0299 (16)	0.0297 (17)	−0.0079 (15)	−0.0066 (15)	−0.0038 (13)
C10	0.040 (2)	0.0471 (19)	0.050 (2)	−0.0118 (17)	−0.0065 (17)	−0.0031 (17)
C11	0.041 (2)	0.0291 (15)	0.0328 (18)	0.0052 (15)	−0.0118 (15)	−0.0065 (13)
C12	0.048 (2)	0.0378 (18)	0.081 (3)	0.0135 (17)	−0.015 (2)	−0.0056 (19)

### Geometric parameters (Å, °)

**Table:**

Br1—C5	1.891 (3)	C3—C8	1.403 (4)
Br2—C7	1.889 (3)	C4—C5	1.376 (4)
O1—C2	1.193 (3)	C4—H4A	0.9500
O2—C2	1.318 (4)	C5—C6	1.370 (4)
O2—C1	1.444 (4)	C6—C7	1.387 (4)
O3—C9	1.209 (3)	C6—H6A	0.9500
O4—C11	1.207 (4)	C7—C8	1.383 (4)
N1—C11	1.400 (4)	C9—C10	1.485 (5)
N1—C9	1.416 (4)	C10—H10A	0.9800
N1—C8	1.439 (3)	C10—H10B	0.9800
C1—H1A	0.9800	C10—H10C	0.9800
C1—H1B	0.9800	C11—C12	1.495 (4)
C1—H1C	0.9800	C12—H12A	0.9800
C2—C3	1.492 (4)	C12—H12B	0.9800
C3—C4	1.397 (4)	C12—H12C	0.9800
			
C2—O2—C1	115.9 (3)	C8—C7—C6	121.9 (3)
C11—N1—C9	125.7 (2)	C8—C7—Br2	120.3 (2)
C11—N1—C8	114.4 (2)	C6—C7—Br2	117.8 (2)
C9—N1—C8	119.8 (2)	C7—C8—C3	118.9 (2)
O2—C1—H1A	109.5	C7—C8—N1	119.2 (3)
O2—C1—H1B	109.5	C3—C8—N1	121.8 (3)
H1A—C1—H1B	109.5	O3—C9—N1	120.5 (3)
O2—C1—H1C	109.5	O3—C9—C10	123.1 (3)
H1A—C1—H1C	109.5	N1—C9—C10	116.3 (2)
H1B—C1—H1C	109.5	C9—C10—H10A	109.5
O1—C2—O2	123.0 (3)	C9—C10—H10B	109.5
O1—C2—C3	124.9 (3)	H10A—C10—H10B	109.5
O2—C2—C3	112.0 (3)	C9—C10—H10C	109.5
C4—C3—C8	119.5 (3)	H10A—C10—H10C	109.5
C4—C3—C2	119.9 (3)	H10B—C10—H10C	109.5
C8—C3—C2	120.5 (2)	O4—C11—N1	118.5 (3)
C5—C4—C3	119.2 (3)	O4—C11—C12	121.8 (3)
C5—C4—H4A	120.4	N1—C11—C12	119.6 (3)
C3—C4—H4A	120.4	C11—C12—H12A	109.5
C6—C5—C4	122.5 (3)	C11—C12—H12B	109.5
C6—C5—Br1	118.1 (2)	H12A—C12—H12B	109.5
C4—C5—Br1	119.3 (2)	C11—C12—H12C	109.5
C5—C6—C7	117.9 (3)	H12A—C12—H12C	109.5
C5—C6—H6A	121.0	H12B—C12—H12C	109.5
C7—C6—H6A	121.0		
			
C1—O2—C2—O1	4.5 (5)	Br2—C7—C8—N1	2.8 (4)
C1—O2—C2—C3	−178.0 (3)	C4—C3—C8—C7	−0.4 (4)
O1—C2—C3—C4	156.5 (3)	C2—C3—C8—C7	179.1 (3)
O2—C2—C3—C4	−21.0 (4)	C4—C3—C8—N1	175.9 (3)
O1—C2—C3—C8	−23.1 (5)	C2—C3—C8—N1	−4.6 (4)
O2—C2—C3—C8	159.5 (3)	C11—N1—C8—C7	85.5 (4)
C8—C3—C4—C5	1.1 (4)	C9—N1—C8—C7	−97.8 (3)
C2—C3—C4—C5	−178.5 (3)	C11—N1—C8—C3	−90.8 (3)
C3—C4—C5—C6	−0.9 (5)	C9—N1—C8—C3	85.9 (4)
C3—C4—C5—Br1	179.7 (2)	C11—N1—C9—O3	5.9 (5)
C4—C5—C6—C7	0.0 (5)	C8—N1—C9—O3	−170.3 (3)
Br1—C5—C6—C7	179.5 (2)	C11—N1—C9—C10	−173.9 (3)
C5—C6—C7—C8	0.6 (5)	C8—N1—C9—C10	9.9 (4)
C5—C6—C7—Br2	−179.0 (2)	C9—N1—C11—O4	−168.9 (3)
C6—C7—C8—C3	−0.4 (5)	C8—N1—C11—O4	7.5 (4)
Br2—C7—C8—C3	179.2 (2)	C9—N1—C11—C12	14.0 (5)
C6—C7—C8—N1	−176.8 (3)	C8—N1—C11—C12	−169.6 (3)

### Hydrogen-bond geometry (Å, °)

**Table:**

*D*—H···*A*	*D*—H	H···*A*	*D*···*A*	*D*—H···*A*
C1—H1A···O4^i^	0.98	2.44	3.404 (4)	168.
C6—H6A···O4^ii^	0.95	2.46	3.237 (4)	140.

Symmetry codes: (i) −*x*+1, −*y*+1, −*z*+1; (ii) −*x*+1, −*y*+1, −*z*.
